# The Genomic Sequence of the Oral Pathobiont Strain NI1060 Reveals Unique Strategies for Bacterial Competition and Pathogenicity

**DOI:** 10.1371/journal.pone.0158866

**Published:** 2016-07-13

**Authors:** Youssef Darzi, Yizu Jiao, Mizuho Hasegawa, Henry Moon, Gabriel Núñez, Naohiro Inohara, Jeroen Raes

**Affiliations:** 1 Department of Bioengineering Sciences, Microbiology Unit, Vrije Universiteit Brussel, Brussels, Belgium; 2 Center for the Biology of Disease, VIB, Leuven, Belgium; 3 Department of Microbiology and Immunology, Rega Institute, KU Leuven, Leuven, Belgium; 4 Department of Pathology, University of Michigan, Ann Arbor, Michigan, United States of America; 5 Comprehensive Cancer Center, University of Michigan, Ann Arbor, Michigan, United States of America; Columbia University, UNITED STATES

## Abstract

Strain NI1060 is an oral bacterium responsible for periodontitis in a murine ligature-induced disease model. To better understand its pathogenicity, we have determined the complete sequence of its 2,553,982 bp genome. Although closely related to *Pasteurella pneumotropica*, a pneumonia-associated rodent commensal based on its 16S rRNA, the NI1060 genomic content suggests that they are different species thriving on different energy sources via alternative metabolic pathways. Genomic and phylogenetic analyses showed that strain NI1060 is distinct from the genera currently described in the family *Pasteurellaceae*, and is likely to represent a novel species. In addition, we found putative virulence genes involved in lipooligosaccharide synthesis, adhesins and bacteriotoxic proteins. These genes are potentially important for host adaption and for the induction of dysbiosis through bacterial competition and pathogenicity. Importantly, strain NI1060 strongly stimulates Nod1, an innate immune receptor, but is defective in two peptidoglycan recycling genes due to a frameshift mutation. The in-depth analysis of its genome thus provides critical insights for the development of NI1060 as a prime model system for infectious disease.

## Introduction

The oral cavity of animals is host to several hundreds of bacterial species, collectively known as the oral microbiota. The healthy oral microbiota plays an important role in maintaining host health by protecting it from invasions by pathogenic species. However, under certain circumstances some of the commensal members, namely pathobionts, can cause disease after dysbiosis, the disruption of healthy microbiota [1–3]. One example of these so-called ‘pathobionts’ is the bacterium NI1060, which plays an important role in the development of murine periodontitis in a ligature-induced model [4]. In the model, placement of the ligature between the molars damages the gingival epithelium and induces oral dysbiosis at the damaged site, leading to resorption of alveolar bone adjacent to the damaged site, in a bacteria-dependent fashion [4], where strain NI1060 dramatically accumulates in the oral cavity [4]. NI1060 induces periodontitis by stimulation of Nod1, an innate immune receptor that recognizes small peptidoglycan-like molecules containing D-γ-glutamyl-*meso*-diaminopimelic acid (iE-DAP) derived from bacteria [4,5]. Like *Aggregatibacter actinomycetemcomitans* (Aa), a bacterium that is associated with the development of aggressive periodontitis in humans [6], it releases high amounts of unidentified iE-DAP-containing molecules that stimulate Nod1 [4]. However, the mechanisms are still unknown.

Monocolonization of NI1060 in germ-free mice is sufficient for its accumulation in the oral cavity and for the induction of alveolar bone loss at the ligature-damaged gingival site [4]. Moreover, other commensals do not accumulate at the damaged gingival site of specific-pathogen free (SPF) mice[4], whereas NI1060 does, suggesting that it possesses unknown mechanisms to out-compete other commensals at these sites. Unraveling the genetic makeup of NI1060 could help us understand these mechanisms at the molecular level and shed light on new preventive strategies against periodontitis. Here we report the complete genomic sequence of NI1060, compare it to several members of the oral microbiota, investigate its taxonomic position in the *Pasteurellaceae* family, and find several genes that could be involved in the regulation of dysbiosis and pathogenicity.

## Materials and Methods

### Genomic sequencing and sequence analysis

NI1060 was grown in Brain-heart infusion medium (BHI) and genomic DNA was isolated as described in [4]. NI1060 genomic DNA was sequenced by combining the Illumina HiSeq 2000 platform as described in [4] and the PacBio RS technology. The high quality Illumina paired-end reads (read length = 51, insert size = 200, total bases = 6163075388) were subsampled by a factor of 14 then assembled into contigs using the SPAdes genome assembler (v.3.1.1) [7]. The resulting contigs (size > 100bp) were then placed into 1 scaffold (size > 400bp) using SSPACE-long-reads (v.1.1) [8] and corrected PacBio Continuous Long Reads (CLR). Gaps in scaffolds were closed iteratively using PBJelly (v.14.9.9) [9] and GapFiller (v.1.10) [10]. The final assembly was automatically improved using Pilon (v1.8) [11] and consists of 2,553,982 bps. The genome sequence was annotated using the RAST annotation server (v.2.0) [12]. Search for common genes with its closest phylogenetic neighbor *Pasteurella pneumotropica* (Pp), Aa and *Escherichia coli* (Ec) was performed using Reciprocal Smallest Distance [13]. The predicted features were visualized in a genomic context using DNAPlotter [14]. The domain structures of the ORFs were predicted by PFAM. Synteny scores were calculated by Quota synteny alignment [15]. The bacteriophage regions were identified by PHAST [16]. For phylogenetic assignment, all *Pasteurellaceae* genomes (finished or permanent draft) were selected from IMG (v.400) [17] to construct 16S rRNA and marker gene trees. For the former, 16S rRNA gene sequences were extracted using an in-house Biopython [18] script that selects the longest of the predicted 16S sequences in a genome and discards sequences smaller than 1200 bases. The selected 16S rRNA gene sequences were refined (using NCBI’s BLASTN [19] on the rRNA_typestrains database) to replace poorly predicted sequences by higher quality sequences from GenBank or SILVA. The refined sequences were aligned using MUSCLE (v3.8.31) [20] with default parameters, then the tree was constructed using FastTree (v2.1.8) [21] with the following arguments: FastTree -nt -gtr -gamma -bionj -slownni -mlacc 2 -spr 4. The procedure described here is similar to the workflow of Phylophlan (0.99) [22] which was used to construct the concatenated tree based on 400 universal proteins. For this purpose, we edited the MUSCLE section of Phylophlan to allow 16 iterations (default in MUSCLE) for the refinement of the multiple sequence alignment instead of only 2, to use the WAG substitution model of sequence evolution, and to compute the tree likelihood under the gamma model with 20 rate categories instead of the CAT model. Trees were displayed using iTol [23]. Phylogenetic matrices were generated using the -makematrix option in FastTree (S1 Table).

## Results

### Genome analysis indicates that NI1060 is a novel member of the *Pasteurellaceae* family

**Fig 1:**
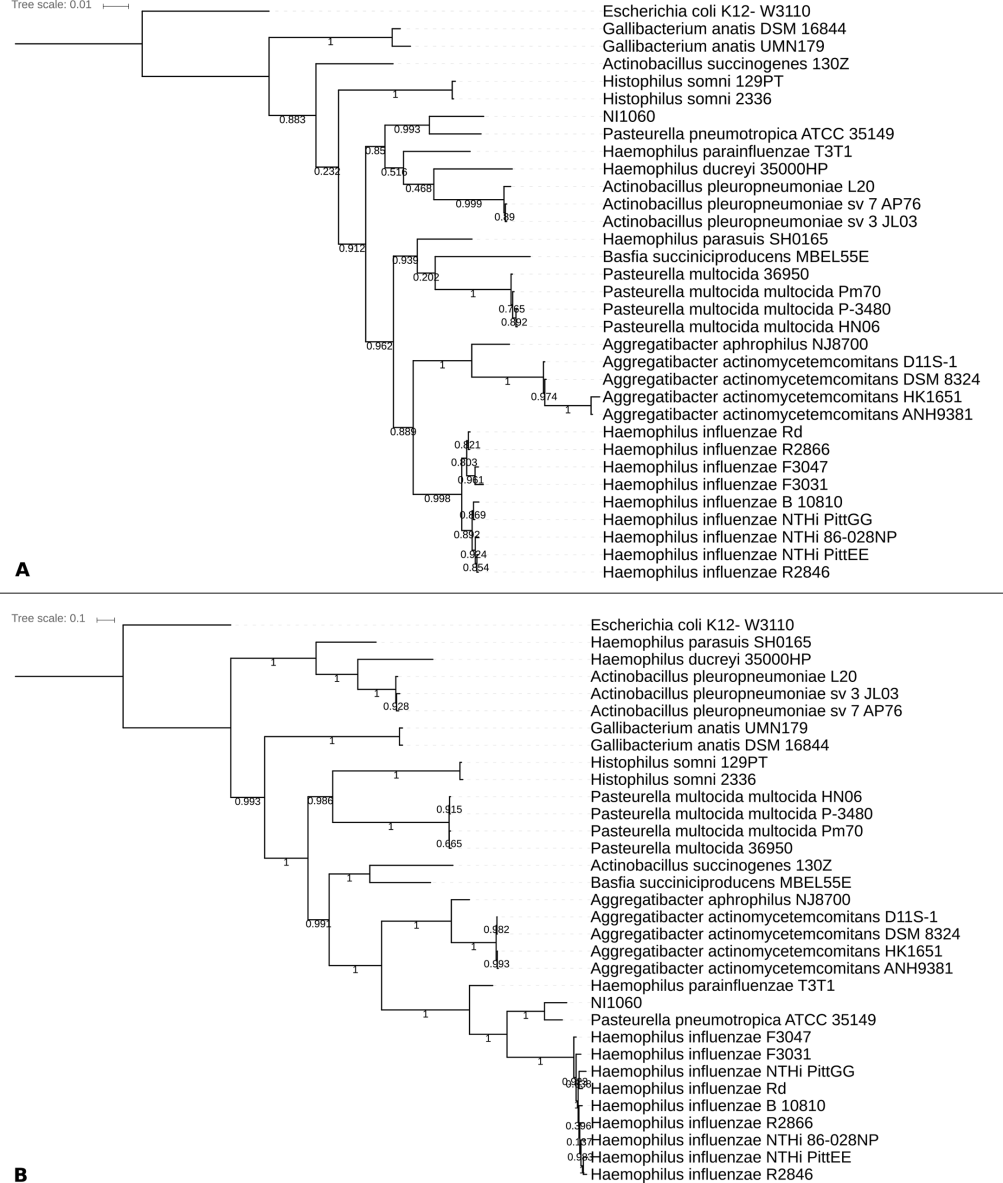
Phylogenetic placement. Phylogenetic placement of NI1060 using the 16S gene (A) and a set of 400 most conserved bacterial genes using Phylophlan (B). The scale indicates the number of nucleotide or amino acid substitutions per site. Both methods show that NI1060 is closest to *P*. *pneumotropica ATCC 35149* but with a branch length suggesting that NI1060 represents a novel species.

**Fig 2:**
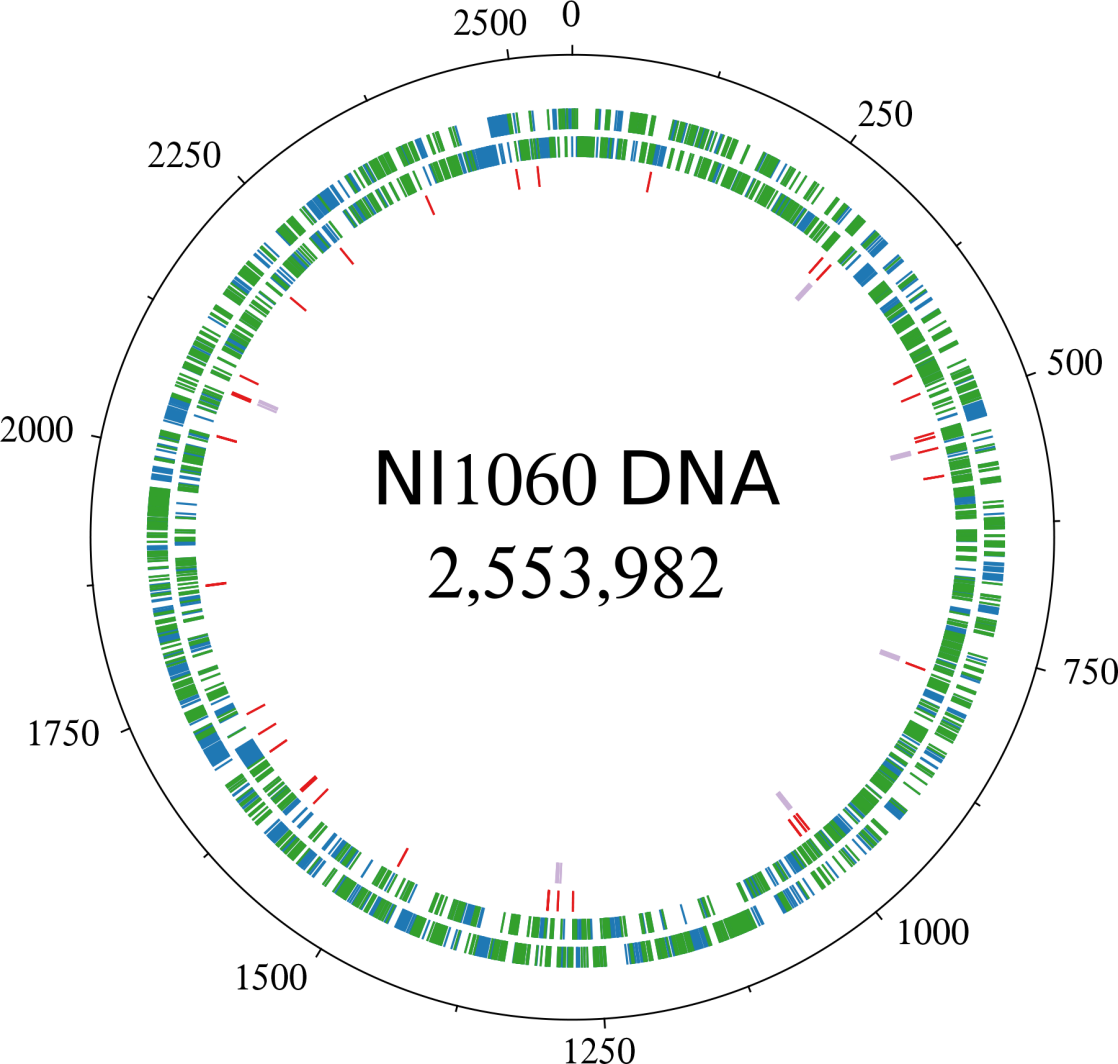
Comparative analysis between NI1060 and *P*. *pneumotropica* ATCC 35149. The putative protein-coding genes on forward (outside) and reverse (middle) strands are show by blue bars. Red bars represent tRNA and purple bars represent rRNA. The protein-coding genes, which are conserved in the related bacterium *P*. *pneumotropica ATCC 35149*, are indicated by green bars.

The genome of NI1060 is comprised of 1 genomic scaffold totaling 2,553,982 bp in length and contains 2,478 predicted protein-encoding genes. Its GC content is 40.3%, which is similar to the average GC content of the *Pasteurellaceae*, but its size (2.6 Mbp) is slightly larger than the average species of this family (2.2 Mbp, data obtained from IMG (v.400)). In addition, it contains six copies of the 16S ribosomal RNA gene with high similarity to those of Pp strains such as T087011-V2, Q480011-V1 (99.5% identical; near-complete sequences of 1517 bp) and ATCC 35149 (96.4% identical; full sequence). This Genome Project has been deposited at DDBJ/EMBL/GenBank under the accession PRJNA288779, biosamples SAMN03801592 and SAMN03840806. Phylogenetic analysis showed disagreement between the 16S rRNA (LogLk = -6729.367) and the concatenated trees (LogLk = -121259.803), which has been previously reported for the *Pasteurellaceae* family [24–26]. Only the concatenated tree recovers the two major clades previously observed in the *Pasteurellaceae* and largely agrees with the marker gene trees reported in recent studies [24,26,27]. In addition, the concatenated tree has a minimum support value of 0.986 for the major branches while the 16S rRNA gene tree shows smaller support values overall with the smallest value of 0.202, meaning that the former is more reliable and underscores again the improved resolution provided by concatenated trees of several universal genes [22,26,28]. However, both phylogenetic trees (16S rRNA and concatenated marker genes) positioned NI1060 next to Pp strain ATCC 35149 on the same branch and phylogenetically well separated from other *Pasteurella* species (**Fig 1**) suggesting they might represent a novel genus. Moreover the branch length suggests that NI1060 represents a novel species which we subsequently verified by calculating several metrics for species delineation using the JSpecies software [29]. All these metrics (ANIb = 86.83%, ANIm = 87.88%, Tetra = 0.98766) fell below the species boundary threshold and thus confirmed our finding. Furthermore, 635 of its 2478 predicted genes (25%) are not present in the genome of Pp strain ATCC 35149 (**Fig 2**) which supports the hypothesis that NI1060 is a species different from Pp which also colonizes the oral and respiratory tracts of rodents [30]. Consistent with the latter finding, NI1060 and Pp behave differentially in that NI1060, but not Pp, accumulates and becomes dominant at the day 10 damaged-ligature sites in 20 tested mice (Figure S2D in [4]). All these findings, suggest that NI1060 therefore represents a novel species of a new genus within the family of *Pasteurellaceae*. A detailed summary of genomic and gene content differences can be found in S2 Table.

### NI1060 possesses unique metabolic pathway genes

The lifestyle of NI1060 suggests that proliferation of this organism may require certain host factors including nutrients from damaged tissue. Consistently, NI1060 was unable to grow in M9 minimal medium (data not shown) and the analysis of its genomic sequence shows that it could grow on host-derived nutrients, via metabolic pathways (e.g. host tissue breakdown) that are not found in any *Pasteurellaceae* member (**S3 Table**). For example, some *Pasteurellaceae* species, such as *Histophilus somni* [31], lack thiamine and pantothenic acid transport systems and genes for the thiamine synthetic pathway. NI1060 harbors these metabolic pathways, but lacks the Pan operon for *de novo* synthesis of pantothenic acid. It also carries genes for the utilization of mono- and di-saccharides like galactose, sucrose, lactose, mannose, maltose, and trehalose, but not of ribitol and cellobiose (**S3 Table**). Like other *Pasteurellaceae* species, it possesses complete gene sets of core biochemical pathways such as glycolysis and gluconeogenesis [4] (also see **S2 Table**) but also has genes for the phosphoglycerate transport system (ORFs 617–620) and the lysine antitransporter system (ORFs 2074, 2075), unlike some *Pasteurellaceae* species(**S3 Table**). Moreover, NI1060 specifically possesses 3 orphan YncD homologues (ORFs 2024–2026) that are presumably involved in the import of unidentified nutrient(s).

Consistent with the fact that NI1060 is a facultative anaerobe, its genome contains several genes that confer the ability to grow under both anaerobic and aerobic conditions. These include genes encoding catalases for aerobic conditions(*kat*, ORF194), periplasmic nitrate reductase system (*nap*, ORFs 1362–1368) and fumarate reductase (*frd*, ORFs 1757–1758) which supports anaerobic growth in the presence of the respective substrates [32,33]. In addition, the orthologues (*cyd*, ORFs1011, 1914) of cytochrome c uniquinol oxidase supports its growth under aerobic conditions, and the orthologues of anaerobic regulatory proteins HlyX/ORF2421, ArcA/ORF1543 and NarP/ORF765 are also found. In contrast to some other Pasturellaceae species including Pp, Aa, and *Haemophilus influenzae* (Hi) [34] that do not possess citrate synthase, aconitase, and isocitrate dehydrogenase (encoding a partial TCA cycle) NI1060, like *P*. *multocida* Pm70, harbors the operon that encodes these enzymes (ORFs260-262) and all other genes important for this process. However, it lacks genes encoding malate synthase and isocitrate lyase which are involved in the glyoxylate cycle, like some *Pasteurella* and *Mannheimia* members including *P*. *multocida* Pm70 (**S3 Table**). It also contains all critical operons for the synthesis of 20 amino acids, purine and pyrimidine nucleotides. This contrasts with Hi and other *Pasteurellaceae* species that require external arginine and uracil as nitrogen sources, whereas external glutamine and cysteine are required for growth in minimal condition medium [35,36]. In addition, Aa and Pp lack phosphoribosylformylglycinamidine synthase which is involved in the *de novo* biosynthesis of purines [35,36]. As for iron, one of the most critical resources for the growth of bacteria [37], NI1060, like other *Pasteurellaceae* species, possesses six iron transport systems including ABC type transporters and iron storage system such as ferritin (**S2 Table**).

Via the host Nod1 receptor, NI1060 stimulates the recruitment of neutrophils [4] that produce oxygen radicals, which some bacteria such as *Salmonella* species can use as an energy source via the tetrathionate utilization (ttr) system [38]. However, NI1060 does not possess the crucial ttr orthologues of *Salmonella*. Therefore, it is unlikely that the accumulation of NI1060 at neutrophil-rich damaged gingival sites is due to increased availability of tetrathionate, suggesting that NI1060 may utilize other energy sources for its accumulation in the oral cavity.

### Repeats, phage-like elements and immunity

Close inspection of the NI1060 genome revealed the presence of several repetitive sequences including those found in transposons and other genes whose details are described below. NI1060 possesses eight genes homologous to ISH50-type transposons and one homologous to the IS1595-type. It also has one mu-type transposon and two retrotransposons (**S4 Table**). Because mu-type transposons are commonly found in other *Pasteurellaceae* species, they are likely to mediate parallel gene transfer and evolution of NI1060 and associated species.

In terms of resistance to bacteriophages, seven regions in the NI1060 genome (≈ 6.9% of the genome) are associated with bacteriophage-like sequences (**Table 1**), similar to those of bacteriophage S1249 in Aa D11S-1, mu/lambda-type bacteriophages, bacteriophages P4 and CP4-57. These phage-related sequences could confer immunity against related bacteriophages such as lysogens. In addition to these, NI1060, like other bacteria, possesses a clustered regularly interspaced short palindromic repeat region (CRISPR) and restriction systems for immunity against pathogens such as phages. The CRISPR system [39] is found in between ORF 1819 and 1822. We also found type I to III restriction systems, which also protect bacteria from bacteriophage infection. Importantly, the homologues of NI1060 type I restriction operons t1a (ORFs 2/2472-2475) and *t1b* (ORFs 276–278) and type III restriction operon *t3a* (ORFs 2223–2225) exist in the genomes of Aa and Hi, but not in that of Pp. In addition, the homologue of its type II restriction operon *t2a* (ORFs 683–684) is not present in the genomes of Hi, Pp and Aa (**S3 Table**). Moreover, the transposon element (position 962862–983841) was found to disrupt the *comM* gene, which is required for efficient bacteriophage recombination [40]. These observations suggest that NI1060 possesses several mechanisms for protection against phage infection.

**Table 1:** Bacteriophage location and annotation.

Region	Region length	Completeness	Score	#CDS	Region position	Possible phage	GC%
**1**	38.3Kb	intact	130	32	504023–542401	PHAGE_Shigel_SfIV_NC_022749	41.11%
**2**	22.1Kb	incomplete	20	16	1667697–1689798	PHAGE_Aggreg_S1249_NC_013597	38.99%
**3**	32.9Kb	intact	150	49	1680840–1713790	PHAGE_Vibrio_pYD38_A_NC_021534	40.12%
**4**	27.8Kb	questionable	70	27	2275583–2303450	PHAGE_Entero_mEp237_NC_019704	39.99%
**5**	54.1Kb	intact	120	77	2434453–2488582	PHAGE_Haemop_Aaphi23_NC_004827	39.92%

One hypothesis for the mechanism of induction of bone loss by NI1060 could be via the cleavage of peptidoglycan by lysozyme (muramidase). This cleavage is expected to produce Nod1 ligand molecules, which are critical for the induction of alveolar bone loss in the ligature model of periodontitis [4,5,41]. Even though NI1060 possesses bacteriophage-related loci with two ORFs homologous to peptidoglycan lysozymes, (1lyz/ORF2375 and 5lyz/ORF1616), we found no evidence of phage-mediated lysis of bacteria under several culture conditions tested (data not shown), suggesting the presence of lysozyme inhibitors. Therefore, the presence of 1lyz and 5lyz could not explain why NI1060 releases high amounts of Nod1 ligand molecules.

### LPS/LOS and polysaccharide structures of NI1060

The structures of lipopolysaccharide/oligosaccharide (LPS/LOS) and capsular polysaccharide are important for resistance against host immunity and dehydration [42,43]. While the structure of the Lipid A portion of LPS/LOS is a critical determinant for TLR4/MD2-mediated immune responses, other polysaccharide portions including the O-region of LPS are critical for recognition by both the innate and acquired immune receptors [43,44]. We found that the NI1060 genome harbors the tetraacyldisaccharide 4'-kinase (lpxK/ORF171), an important enzyme for the production of the phosphorylated lipid A moiety, which is crucial for TLR4 stimulation (**Table 2**). Both NI1060 and Aa, but not Pp nor Hi, possess an operon, that is putatively involved in lipid synthesis of the outer membrane (ORFs 860 to 892), which includes the 1-acyl-sn-glycerol-3-phosphate acyltransferase homologue ORF890, suggesting that this operon is potentially involved in acyl modification of lipid A.

**Table 2:** List of NI1060 genes potentially involved in dysbiosis and pathogenicity.

Function	Domain	Gene ID# (ORF#)
**Hemolysin**		hemolysin (1714, 1717)
**Adhesin**	YadA (anchor)	yadA1(310), yadA7a(1772), yadA2(1293), yadA3(1236), yadA4(1144), yadA5(1083), t6ss5K(990)
	YadA_head	yadA1(310), yadA7c(1769), t6ss5K(990)
	YadA_stalk	yadA1(310), yadA7b(1770), yadA2(1293), yadA4(1149), yadA5(1083), t6ss5K(990)
	Fil_haemagg	cdiA(1943)
	Fil_haemagg_2	CdiA(1943), hlyA(622), cdilA(2445), cdiA2(1954), CdiA3(1956)
	Haemagglutination activity domain	cdiA(1943), hlyA(622), cdilA(2445)
	Type V (ESPR)	yadA1(310), hlyA(622), cdilA(2445), yadA5(1083), 1149, 1293, 1945
**Secretion system**	Type Vb (two factor)	cdiB(1942), hlyB(624), cdilB(2446)
	Type Va (autotransporter)	ag43L(25), picA(826), perT(1077)
	Type VI	rhs1(80–85), t6ss1(285–309), t6ss2(323–326), t6ss3(1453–1451), t6ss4(1404–1406), t6ss5(983–991)
**Bacteriocin**	colicin-type nuclease	cdiA(1943)
	PT-VENN	cdiA(1943), cdiA2(1946), cdiA3(1948)
	Pfam-B_9947	cdiA(1943)
	FhaB(Pfam-B_7836)	cdiA(1943)
	Fido (Fic)	cdilA(2442, 2443)
	Haemocin synthesis protein (Colicin V production protein)	cvpA(1885)
**Flp operon**		flp(1409–1423)
**Type IV pilus**		598–606
**Nod1 ligand recovery**		mppA(230),oppA(1115)
**Nod1 ligand processing**		ampD(598), ampG(1686)

All essential proteins for LPS/LOS core synthesis, including Wzx flippases, Wzy polymerases and WaaL ligases, were encoded in the NI1060 genome. These essential proteins include synthetic enzymes of phospho-KDO (ORF1387, ORF220) and heptose (ORF1388; ORF1320 and ORF1319; ORF1339 to ORF 1341). Interestingly, two sets of loci for putative outer region-associated synthesis were found in the NI1060 genome: *oas2* (ORF1743 to ORF1752) and *oas1* (ORF68 to ORF75) (**S1 Fig**). Both loci are unique to this particular group of bacteria. *oas1* contains two operons (ORFs 68–72 and 73–75) that might be involved in capsular polysaccharide synthesis because of the high similarity of *yvfF*/ORF74 to *Bacillus* exopolysaccharide synthesis gene *epsO* [43].

We also found an additional locus, (*ias*, ORFs 869–891), containing putative genes for synthesis of the lipid and the inner carbohydrate regions. NI1060 has two sets of lipid synthesis proteins, including 3-oxoacyl-(acyl-carrier-protein) synthase, suggesting that it has heterogeneous acyl composition within the lipid A. Both *oas1* and *oas2* loci encode Wzx flippase and glycosyltransferase homologues, but no polysaccharide ligase gene was found in *oas2* suggesting that *oas1* is involved in the synthesis of the outer core region, whereas *oas2* may be involved in the synthesis of carbohydrate branches. The latter includes *wbaP* for galactose export, implying that the first component of polysaccharide metabolism is galactose. It also possesses *rmlB* which encodes the synthetic enzyme of L-Rhamnose precursor, suggesting that the second and distal component of polysaccharide is L-Rhamnose. Importantly, the third predicted component of the polysaccharide is N-acetylneuramine due to the presence of *neuA*, *neuB*, *neuC*, *neuD* and *siaA* in *oas2*. This is important because sialylation of the LPS/LOS outer region in Hi and other bacteria is known to be crucial for resistance to complement attack [42] and potentially, for reducing TLR4 activation [43]. *Oas2* also contains two putative glycosyltransferases (orfO/ORF1750 and kfiC/ORF1751), which mediate modification of the outer chain, although their substrate specificities cannot be predicted due to the low homology to known glycosyltransferases. Furthermore, NI1060 encodes an uncharacterized glycosyltransferase (ORF1455), which could further modify the core structure of LOS. The homologous loci to NI1060 *oas1* and *oas2* were also found in *Pasteurellaceae* species including Hi encapsulated and non-capsulated strains, indicating their potential implication in the production of the outer core of the LOS R region but not the capsule. Importantly, we found that the gene organization of *oas1* and *oas2* in NI1060 is different from all *Actinobacillus* or *Pasteurella* commensals. This suggests that the LPS/LOS structures of NI1060 are different from these commensals and likely contribute to different sensitiveness to host immunity including the complement system.

### Putative virulence factors in NI1060

NI1060 is sufficient to induce alveolar bone loss after ligature-induced host damage in a mouse periodontitis model and belongs to the *Pasteurellaceae* family like Aa, a bacterium associated with aggressive periodontitis in humans [45]. To gain insight into the mechanism by which NI1060 induces alveolar bone loss at host damaged sites, we searched its genome for known factors of bacterial pathogenicity. We found that it lacks the *ltxA* gene, a released factor which is important to induce host cell cytotoxicity by Aa [45]. However it contains fragmented ORFs (ORF1713-ORF1717) similar to *pnxIIIA* of Pp that might be linked to the pathogenicity of Pp [46,47]. Consistent with the latter, we found that NI1060 does not exhibit significant cytotoxic activity against several human and mouse cells tested, when compared to Aa [4]. We found no orthologues of *Porphyromonas gingivalis* gingipains, *Treponema denticola* dentilicins, *Tannerella forsythia* HrtH proteases and toxins, except Cdi proteins that are described later. This is consistent with the fact that oral colonization of NI1060 does not cause damage of the gingival epithelium or alveolar bone loss in the absence of ligature-induced host damage [4]. Interestingly, NI1060 possesses InlA (ORF1779), a homologue of *Listeria monocytogenes* internalin A, which mediates internalization of *Listeria monocytogenes* into epithelium via interaction with E-cadherin [48]. The function of this protein is unclear because we have no evidence for intracellular localization of NI1060 but one possibility is the attachment to host cells on epithelium or in the damaged tissues.

PFAM analysis showed that several ORFs in the NI1060 genome are homologous to essential proteins for bacterial competition and pathogenicity. For instance, we found homologs for the machinery of several secretion systems including type I, II, V and VI, but not III and IV. Importantly, many proteins that are translocated across the outer membrane by type V and VI secretion systems (T5SS and T6SS) are known to be involved in bacterial competition and interactions with eukaryotic cells [49,50]. In NI1060, there are three proteins for the autotransporter Va-type system, five proteins for two factor type V (Vb) secretion system (**Table 2**), and six loci for T6SS. All T5SS proteins, except Ag43L/ORF25, possess either one or a combination of haemagglutination activity domains, YadA domains, and filamentous haemagglutination domains (**Table 2**). *Cdi*, one of the T5SS-related loci, is homologous to those involved in contact-dependent inhibition (CDI) in proteobacteria including Ec and *Burkholderia pseudomallei* [51]. It is an "orphan"-type locus that contains two additional CdiA C-terminal region-CdiI modules [51]. One, Ag43L, is similar to Ec autotransporter Ag43, which is involved in adhesion and virulence [52]. Although Ag43L does not contain the AidA adhesion domain like Ag43, it contains an uncharacterized conserved domain (amino acid position 1–220) that is homologous to other putative adhesins and autotransporter proteins including *Oscillatoria nigro-viridis* PCC 7112 (YP_007114496) and Ec 536 (YP_669320), suggesting that NI1060’s Ag43L might also be involved in adhesion and virulence through a novel domain.

NI1060 harbors seven loci encoding proteins that are translocated across membranes via T6SS. The locus *t6ss1* (ORFs285-309) is composed of three operons that encode a full set of secretion system components and effectors including VgrG, Hcp, ImpB ClpV, EvrB and FHA domain proteins [50], and four loci encode at least 9 effector proteins. However, we found that ORF298 the orthologue of *vasE*, which is critical for a functional T6SS [53], has one frame-shift mutation. This, together with the fact that the enforced colonization of NI1060 induces periodontitis phenotype at the ligature-damaged site [4], indicates that T6SS is not required for the disease development.

NI1060 also possess the *tad* locus encoding Flp fimbriae (ORFs 1409–1423), which is critical for biofilm formation, colonization and virulence in Aa [45,54]. Furthermore, in a rat model for periodontal disease, *flp-1*/ORF1409 and *tadA*/ORF1416 mutants of Aa showed no evidence of bone loss, demonstrating that the *tad* locus is essential for virulence in Aa [55]. In addition, NI1060 possess a locus encoding the type IV pili (ORFs598-606), known to be important for interactions with other bacteria and host cells [56] (**S3 Table**). Although NI1060 is Nod1-stimulatory, it lacks a type IV secretion system which is used by *Helicobacter pylori to* inject Nod1 ligands into host cells [57]. Yet, many bacteria including NI1060 are known to stimulate host cells without active injection of microbial ligands [58], probably due to the presence of active transport systems present in host cells [59]. Concerning the secretion of Nod1 ligand molecules, we found two homologues (OppA/ORF230 and OppA/ORF1115) of Ec MppA, which *are* important for the salvage of Nod1 ligand molecules and the release of Nod1 ligands during peptidoglycan remodeling [41,60]. Interestingly, the associated Opp Transport system contains a frameshift mutation in the *oppC* gene (validated by Sanger sequencing; data not shown). Therefore, it is likely that the high amount of Nod1 ligands released by NI1060 is due to the lack of a functional OppABCDF system required for peptidoglycan recycling. Another frameshift mutation was found in the oligopeptide transporter gene (opt/ORF856-857) (validated by Sanger sequencing; data not shown), also reported to be involved in the uptake of iE-DAP into the cytosol [59], and thus this frameshift mutation could be involved in Nod1 stimulation by NI1060. In contrast, wild type copies of *oppC* and *opt* are found in Pp. We also found that NI1060 has YafK/ORF631, a homologue of Campylobacter Pgp2, which can affect Nod1-stimulatory activity of peptidoglycan-related molecules [61], and therefore NI1060 YafK may be also involved in Nod1 stimulation (**S3 Table**).

## Discussion

In this study, we have determined and analyzed the complete NI1060 genome, a novel model organism for oral pathobiont research. Although its 16S rRNA phylotype is highly similar (99.5%) to partial 16S sequences of Pp T087011-V2 and Q480011-V1, it is only 96% similar to that of Pp strain ATCC 35149. Moreover, 25% of NI1060’s genes are absent in Pp ATCC 35149, and thus, NI1060 potentially represents a novel species of a new genus as illustrated by ANI analysis and phylogenetic reconstruction as well. In contrast to Pp which is a dominant member of the healthy murine-oral microbiota, NI1060 is a low abundant member but accumulates and becomes dominant at damaged gingival tissues (based on 20 mice, as shown in the Supplemental Experimental Procedures in [4]). For this reason, we performed an in-depth comparison of the genomes of these two related species, and that of human periodontitis-associated Aa, with the ultimate aim of unraveling the mechanisms by which NI1060 accumulates and becomes dominant at the damaged gingival epithelium, and induces alveolar bone loss in the murine periodontitis model. Overall, the two species show different gene syntenies and a considerable variation in their gene pool with regards to metabolism, bacterial and host interactions. Notably, Aa possesses leukotoxin which is important for its cytotoxicity [62], while NI1060 does not, which suggests that NI1060 cannot induce epithelial damage by leukotoxin and might explain why NI1060 can only induce alveolar bone loss in the presence of ligature-induced gingival damage [4,45]. In addition, both Aa and NI1060 induce high Nod1-stimulatory activity leading to the recruitment of neutrophils that activates the secretion of inflammatory cytokines [4]. In activated T cells, proinflammatory cytokines induce the expression of RANKL which plays an essential role in alveolar bone loss during periodontitis development [63]. Indeed, in the ligature-induced periodontitis model, RANKL expression is increased and mature lymphocytes are essential for alveolar bone loss [4]. Also, in Aa infection-induced periodontitis model, Pp is important for T cell activation via the cross reactivity between the Aa Omp29 (OmpA orthologue) and the Pp OmpA. Consequently Pp may play a key role in the induction of lymphocyte activation and alveolar bone loss [64]. Then for the fact that NI1060’s OmpA is 78% and 66% identical to *Pp* DSM 21403 and Aa D7S-1 OmpA proteins, respectively, whereas DSM 21403 OmpA is also 66% identical to Aa D7S-1 OmpA, it will be interesting to test if Aa-induced periodontitis is dependent on NI1060, using GF mice colonized by these two bacteria. Finally, the possibility that NI1060 is capable of triggering both neutrophil recruitment and lymphocyte activation via Nod1 ligand and OmpA, respectively, could also explain why monocolonization of NI1060 is sufficient to induce alveolar bone loss [4].

The current work suggests a model for the induction of periodontitis in humans in which multiple oral bacteria play an important role in alveolar bone loss. These include 1) bacteria that release Nod1 ligands, 2) host-damaging bacteria that damage the epithelial barrier to allow translocation of Nod1 ligands, 3) commensals that provide antigens to activate lymphocytes that induce RANKL expression. These hypotheses could be further tested by colonizing GF mice with NI1060 mutants lacking critical factors for alveolar bone loss, in the presence or absence of a particular set of bacteria of the human healthy oral microbiome.

## Supporting Information

S1 Fig Oas1 and Oas2 loci putatively associated with LPS/LOS and polysaccharide synthesis. Arrows show sizes and orientations of genes. Black and white arrows show Oas and neighbor genes, respectively. The gene names were given from the most homologous genes of other bacteria.
(EPS)

S1 Table Phylogenetic matrices for the 16S and the concatenated tree. (XLS)

S2 Table The list of putative ORFs in NI1060 genome. The ORFs are shown with the size, coding strands, 5' and 3' positions, gene gaps, contig identification, amino acid sequences, closest homologues and similarity score of the indicated species, gene name, description and predicted domain structures by PFAM.
(XLSX)

S3 Table Comparison of genomic features of NI1060 with other bacteria. (XLSX)

S4 Table Transposons. (XLSX)

S5 Table Bacteriophage mapping details. (XLSX)
